# Emerging mechanism-driven therapies for endometriosis: From GnRH antagonists to microbiome and device-based strategies (Review)

**DOI:** 10.3892/mi.2026.341

**Published:** 2026-09-28

**Authors:** Krupa Sara Thomas, Subrahmaniyan Sujitha Lekshmi, Harshitha Gurrala, Mohamed Rayyan Ramzan, Ahmed Khalid Osman, Ziya Sherin

**Affiliations:** 1 Department of Medicine, Faculty of Medicine, Tbilisi State Medical University, Tbilisi 0177, Georgia; 2 Department of Medicine, Faculty of Medicine, School of Health Sciences and Public Health, University of Georgia, Tbilisi 0177, Georgia

**Keywords:** endometriosis, emerging therapies, GnRH antagonists, microbiome, biomarkers, precision medicine

## Abstract

Millions of women of reproductive age suffer from endometriosis, a chronic, estrogen-driven condition that causes pelvic pain, dysmenorrhea and infertility. Conventional therapy focuses on symptom suppression with hormone medications or the surgical excision of lesions; however, it is not curative. Recent developments in the understanding of the pathophysiology of endometriosis have revealed novel immunological and molecular mechanisms that are being used in experimental therapies. The present review outlines developing techniques, including targeted endocrine modulators, epigenetic treatments, anti-inflammatory and immunomodulatory drugs, antioxidants, anti-angiogenic medicines, and microbiome-based therapy. Prominent examples are new oral gonadotropin-releasing hormone receptor antagonists, selective progesterone receptor modulators and aromatase inhibitors that aim to overcome progesterone resistance. Alternative methods, such nutraceuticals and biologic and small-molecule inhibitors (such as metabolic drugs and anti-cytokine therapies) are being explored. Novel supplemental options are provided by device-based neuromodulation and improved surgical techniques (such as advanced laparoscopy, robotic excision, image-guided surgery, nerve-sparing techniques and adhesion barriers). While many of these innovations are promising, current evidence is limited by small studies and disease heterogeneity. Future directions emphasize rigorous trials with standardized outcomes, including fertility measures and combinations of therapies to achieve personalized, long-term relief.

## 1. Introduction

Endometriosis is defined as ectopic endometrial tissue outside the uterus, where the ‘endometrium’ is the mucosal lining of the inner cavity of the uterus (1). Ectopic tissue may occur within the peritoneal cavity, usually involving the ovaries, outer uterine walls, and bladder, but it can also affect the intestines and lungs (2-4).

Of note, >170 million individuals worldwide suffer from endometriosis (2). A recent systematic review and meta-analysis found a pooled prevalence of ~18% (95% CI, 16-20) (5); however, a number of studies still use an estimate of ~10-15% of women of reproductive age (3) and 30-50% of women with infertility (4).

Endometriosis affects ~10% of women of reproductive age worldwide. In selected surgical populations, particularly in women undergoing diagnostic laparoscopy for infertility or chronic pelvic pain, substantially higher detection rates have been reported. According to the review by Smolarz *et al* (3) there is a wide prevalence range (0.1-53%), reflecting differences in patient populations, indications for surgery, and diagnostic approaches rather than the true population prevalence. Of note, ~7% of patients with endometriosis report a family history. In older operative series, endometriosis was detected in ~2% of women at tubal ligation and 17% following ovarian surgery. In addition, endometriosis has been reported in postmenopausal women (3).

While certain research suggests that endometriosis is more common in Asian women and less common in women of African origin than in their Caucasian counterparts, other studies that have examined women of different racial backgrounds with similar symptoms and socioeconomic position have not shown any differences (6). It was also noted that endometriosis was more common in developing countries than in industrialized ones, according to Moradi *et al* (5).

The most common manifestations associated with endometriosis are chronic pelvic pain, painful or heavy menstruation (dysmenorrhea), painful sexual intercourse (dyspareunia) and infertility (4). Symptoms can be particularly severe in adolescents. Among women presenting with dysmenorrhea or dyspareunia, ~70% have been found to have endometriosis in diagnostic or operative series (5). In addition to the clinical manifestations, it can have a notable negative influence on psychological health, often leading to anxiety, depression, impaired social relationships and impaired sexual relationships. Moreover, the financial impact of the disease is substantial, both for individuals and society as a whole, as it contributes to increased health care costs and decreased productivity at work. Endometriosis is an issue of utmost significance from a social and economic, as well as a medical perspective (2-4).

Endometriosis is divided into three main types: Superficial peritoneal lesions (being the most common), ovarian endometrioma and deep infiltrating endometriosis (7-9). Furthermore, the American Society for Reproductive Medicine (ASRM) classification system assigns a scoring scale to categorize the disease as minimal (stage I), mild (stage II), moderate (stage III) and severe (stage IV) (8).

The symptoms of endometriosis can be managed with a variety of modern medical interventions. However, these treatments should be viewed as suppressive and not curative in nature. The optimal regimen is selected based on a variety of parameters, including age, treatment preferences, reproductive plans, disease severity and pain intensity. This is due to the comparable effectiveness of various pharmaceutical treatments in symptom reduction (8).

When endometriosis is suspected in young women prior to the surgical confirmation of lesions, treatments are frequently initiated. Treatments are also provided following surgery in the event that symptoms do not improve or if the illness recurs. The frequently used medications function by altering hormone levels, either by reducing ovarian activity and sex steroid production or by directly targeting steroid receptors and enzymes within the endometrium and ectopic lesions. Examples of these medications include combined oral contraceptives, various forms of progestogens, anti-progestogens, gonadotropin-releasing hormone (GnRH) agonists and antagonists, along with aromatase inhibitors. In addition, it should be noted these medications are associated with adverse effects, such as headaches, nausea, hot flashes and night sweats. Moreover, their contraceptive action may be advantageous for women not seeking pregnancy, whereas this poses a challenge for those desiring fertility (9).

The present literature review aimeds to critically evaluate emerging therapeutic approaches in the management of endometriosis. The searched literature covered publications from 2015 to 2025, encompassing original research articles, randomized controlled trials (RCT), clinical studies, systematic reviews and meta-analyses published in peer-reviewed journals in various databases, such as PubMed, Scopus, Web of Science and ScienceDirect.

## 2. Pathophysiological targets driving emerging endometriosis therapies

Although the precise pathophysiology of endometriosis remains unknown, it is most likely complex, involving immunological, hormonal, genetic and environmental variables (10).

*Epigenetic alterations*. Endometriosis is strongly influenced by epigenetic changes, which include, reversible DNA methylation and histone modifications that alter gene expression without changing the DNA sequence (11). These modifications affect key pathways (hormone synthesis/signaling, immunity and apoptosis) in endometrial cells. Notably, as epigenetic marks are reversible, they present tractable targets (12,13). For example, studies note that ‘epigenetic-based therapies aim to reverse pathological gene expression patterns’ and hold promise for the personalized treatment of endometriosis (13).

In practice, one can envision using DNA methyltransferase or histone deacetylase inhibitors to ‘reset’ aberrant gene expression in ectopic endometrium. The ‘epigenetic hypothesis’ suggests that accumulating epigenetic alterations (some inherited, some acquired via environmental insults such as oxidative stress or toxins) generate new endometriotic clones. Each lesion may then carry a distinct epigenetic profile, explaining the heterogeneity of disease. In sum, the plasticity of the epigenome is being exploited in new therapies aimed at reprogramming endometriotic cells (14).

### Oxidative stress and inflammation

Retrograde menstruation and chronic peritoneal inflammation create a highly oxidative environment in endometriosis. In order to cause endometriosis, endometrial cells must adhere and multiply once they are inside the peritoneal cavity. Elevated levels of reactive oxygen species (ROS), often from iron overload in menstrual blood, activate redox-sensitive pathways, such as NF-κB in macrophages that upregulate pro-inflammatory cytokines, adhesion molecules and growth factors (15). This oxidative microenvironment promotes lesion establishment by enhancing inflammation, extracellular-matrix degradation, angiogenesis and cell proliferation. For example, patients with endometriosis exhibit high levels of peritoneal ROS and low levels of antioxidants, driving a feed-forward cycle of inflammation and tissue growth (16). Chronic cytokine release also ‘sensitizes’ nearby sensory nerve fibers within ectopic endometrial lesions, thereby facilitating the relay of pain impulses to the central nervous system (17). IL-1β and TNF-α directly lower the activation threshold of nerve endings via transient receptor potential vanilloid 1 (TRPV1), causing the release of neuropeptides, such as substance P and calcitonin gene-related peptide (CGRP), as well as neurogenic inflammation (18). Clinically, this accounts for progressive pelvic pain and hyperalgesia. Restoring redox balance is thus a therapeutic goal (16). In experimental models, antioxidants such as N-acetylcysteine, curcumin, melatonin and vitamins C/E have shown benefits, such as reducing oxidative stress, limiting lesion growth and alleviating pain symptoms (18). These findings support trials of antioxidant-based therapies to interrupt the ROS-inflammation cycle in endometriosis.

### Gut microbiota (‘estrobolome’) and endometriosis

Emerging evidence implicates the gut microbiome in the pathophysiology of endometriosis. In particular, the estrobolome (gut bacteria that produce β-glucuronidase and β-glucosidase) can deconjugate estrogen and increase its enterohepatic reuptake. The dysbiosis of the estrobolome may thus raise circulating estrogen levels, fueling ectopic tissue growth and inflammation (15,17,19,20). More broadly, women with endometriosis have an altered gut microbial diversity and composition compared to healthy controls (21). Specific bacterial shifts (for example, the overgrowth of potential pathogens such as *Shigella* and *Escherichia coli*) have been reported in stool samples from patients with endometriosis. These changes in the microbiome may not only contribute to the pathogenesis of the condition, but may also serve as a non-invasive diagnostic biomarker (21). At present, the diagnosis often requires a long period of time, usually between 4-12 years, as symptoms are vague and can mimic other conditions, while the definitive diagnostic method, laparoscopy, remains an invasive operation (19). Indeed, researchers have demonstrated distinct gut microbial and metabolite signatures in endometriosis, and they are actively exploring gut microbiota profiling as a novel screening tool (21).

In the future, therapies that normalize the gut microbiome (probiotics, diet, antibiotics or fecal microbiota transplant) could potentially complement standard treatments by rebalancing the estrobolome and reducing estrogen-driven stimulation (22).

### Angiogenesis and hypoxia

The growth and maintenance of endometriotic lesions critically depend on new blood vessel formation (23,24). Ectopic implants typically exist in a relatively hypoxic milieu, which stabilizes hypoxia-inducible factor-1α (HIF-1α). HIF-1α in turn upregulates angiogenic factors, such as vascular endothelial growth factor (VEGF) to promote neovascularization. In endometriosis, the high expression of HIF-1α has been observed in ovarian endometriomas, and hypoxia strongly induces VEGF, platelet-derived growth factor, placental growth factor and hepatocyte growth factor, all key drivers of angiogenesis (23). According to the genetic ontology assessment of differentially expressed genes, angiogenesis is primarily limited to endothelial cells and pericytes, which suggests that both cells types play a critical role in new blood vessel formation in endometriosis (24). In addition, the high vascularization of these endometrial lesions indicates that targeting angiogenesis may be a viable therapeutic approach (25).

### Neurogenic inflammation and pain sensitization

Severe pain is a hallmark of endometriosis, and it arises from an active neuro-inflammatory process. Endometriotic lesions become densely innervated by sensory and sympathetic fibers (18). Inflammatory mediators released locally, many from activated macrophages, encourage nerve growth and sensitization (18,26). For instance, IL-1β (elevated in lesion fluid) stimulates the overexpression of nerve growth factor and other neurotrophins (27). It has been shown that pro-inflammatory cytokines (IL-1β and TNF-α) can directly sensitize nociceptors through TRPV1 channels. TRPV1-expressing neurons then release substance P and CGRP, driving neurogenic inflammation and further amplifying pain signaling (18). This increased neural infiltration may substantially contribute to increased sensitivity to mild or non-painful stimuli and to the persistence of chronic pain by lowering the activation threshold of sensory nerve fibers. Furthermore, elevated serum prolactin levels may heighten the responsiveness of pain-sensing nerve fibers throughout the body, thereby increasing the susceptibility to developing additional pain disorders (28).

In parallel, the levels of neurotrophic factors are increased: Brain-derived neurotrophic factor (BDNF) and neurotrophin-3 are upregulated in endometriotic tissue. Circulating levels of BDNF are higher in women with endometriosis and are associated with more intense pain, and estrogen is known to drive BDNF/NT3 expression in lesions (18). Collectively, these changes lower the activation threshold of sensory nerves, explaining hyperalgesia and allodynia in endometriosis. Targeting this neuro-immune crosstalk is an emerging strategy.

## 3. Current standard of care

Even though a cure for endometriosis does not currently exist, its symptomatic management is achieved by clinicians with the aid of research and clinical experience. This management is generally divided into pharmacological and operative interventions (9). There are two types of pharmacological treatment: Hormonal and symptomatic. The type of intervention is selected based on the age and reproductive wishes of the patient (3).

The objectives of pharmaceutical treatment are to reduce or eliminate discomfort, prevent the growth and regression of endometrial foci and restore fertility (3). Combined oral contraceptives (COCPs) and progestogens are considered first-line, and the levonorgestrel intrauterine device (LNG-IUD) is an alternative first-line treatment according to international guidelines (29). Progestogen preparations result in reduced levels of GnRH, follicle-stimulating hormone (FSH) and luteinizing hormone; thereby, they suppress the endometrial activity, which leads to pain relief. COCPs stabilize the endometrium by lowering FSH levels, as do LNG-IUDs, and are specifically recommended for patients with severe pain (3). These therapies create a progestogenic, ovulation-suppressive milieu and are recommended as first-line treatment options (30). Dienogest differs from other first-line progestins by the local anti-inflammatory and antiproliferative effects on endometriosis lesions. The long-term use of this medication can reduce the size of endometriomas and prevent recurrence (31). Muzii *et al* (32) reported that dienogest was effective in reducing disease and pain recurrence after endometriosis surgery. The systematic review by Liu *et al* (33) summarized the results of dienogest treatment following surgery compared to no treatment (four studies) or other treatments (GnRH-a, five studies; LNG-IUS, two studies). A lower rate of recurrence was associated with dienogest treatment [odds ratio (OR), 0.14; 95% CI, 0.07-0.26 vs. no treatment, and OR, 0.46; 95% CI, 0.24-0.86 vs. other therapies] (33). In another study, dienogest and COCPs were found to significantly decrease pelvic pain levels when compared to placebo in a randomized clinical pilot trial carried out in Tehran, Iran (2018-2020) (P=0.026); the two active therapy groups did not significantly differ in their ability to reduce pain (34).

The second-line management of endometriosis includes GnRH agonists together with hormone replacement therapy, GnRH receptor antagonists and danazol. These therapies lower the level of estrogen, also termed as hypoestrogenic therapy (30,35). Both agonists (e.g., leuprolide acetate) and antagonists (e.g., elagolix) of the GnRH receptor help lower estrogen levels and ameliorate symptoms; however, agonists initially increase activity, followed by the reduction and inhibition of estrogen production; by contrast, antagonists cause the same effect without causing an initial flare-up (10). The symptomatic management of pain is achieved with the use of nonsteroidal anti-inflammatory drugs (NSAIDs) by decreasing the production of prostaglandins, which are implicated in causing pain in endometriosis (36). The use of physiotherapy in endometriosis also helps in improving pelvic function, lessening discomfort and improving overall quality of life through techniques, such as kinesiotherapy, manual therapy and physical modalities, such as electrotherapy and laser therapy (37). A decrease in pelvic pain (-1.4; 95% CI, -2.7 to -0.1; P=0.036) and dyschezia (-3.5; 95% CI, -5.8 to -1.3; P=0.003), as well as improvements in physical quality of life (3.8; 95% CI, 0.5-7.1; P=0.026) and mental quality of life (5.9; 95% CI, 0.6 to 11.3; P=0.031), were reported following a 3-month psychotherapy intervention with somatosensory stimulation compared with waiting-list controls (38).

Surgical management is indicated when the presumptive therapy is not effective, under the circumstances where there is a lack of efficacy of medical treatment, or when rapid diagnosis and treatment are necessary. This strategy is also employed following an unsuccessful trial of three medications as an economical strategy (30). Surgical management can be classified into conservative and definitive procedures, in which fertility is preserved and compromised, respectively (39). Saunders and Horne (9) summarized that laparoscopic surgery was shown to provide more benefits than laparoscopic diagnosis alone by removing and destroying lesions and reestablishing normal tissue function. It has more effects in reducing pain, particularly in the case of ovarian and deep lesions (OR; 6.58; 95% CI, 3.31-13.10) (9). In a study conducted by Giese *et al* (40), standard care and non-specific acupuncture at home were compared to acupuncture and found to be successful in lowering overall pelvic pain (Hedges' g=1.54; 95% CI, 0.92 to 2.16; three RCTs, n=231, low certainty evidence, P<0.001), menstrual pain (g=1.67; 95% CI, 1.23 to 2.12; one RCT, n=106, moderate certainty evidence, P<0.001) and non-specified pelvic pain (mean difference -2.77; 95% CI, 2.15 to 3.38; two RCTs, n=125, low certainty evidence, P<0.001) at the end of the treatment (40). In another study Wang *et al* (41), anti-Müllerian hormone (AMH) levels were found to be markedly decreased in women with bilateral cysts, cysts >7 cm and stage IV endometriosis 12 months following surgery (the type of surgery did not make a difference) in a study on 171 women. In another study, laparoscopic cystectomy reduces recurrence (9.6-45%), improved spontaneous pregnancy chances (14-54%) and relieved pain; however, this was at the expense of decreased ovarian reserve and potential ovarian failure, particularly following bilateral surgeries. Serum AMH levels in unilateral (194 women) vs. bilateral (124 women) endometrioma excision following 9-12 months were compared; serum AMH levels were considerably lower in the bilateral group (95% CI, 0.63-1.52; P=0.001) (42). There was little heterogeneity among the trials (I²=37%, P=0.20). In the unilateral and bilateral groups, other methods, such as cyst draining with ablation or ultrasound-guided aspiration, were associated with higher recurrence rates and were primarily used to maintain fertility or relieve symptoms (42). The surgical strategy must be balanced between the outcomes while controlling the risk of injury when fertility preservation or optimization is considered. Surgery can result in harm to ovarian reserve and accessibility (43). Assisted reproductive technologies (ART), such as IVF are preferred in women with endometriosis aged >35 years, if the ovarian reserve is low, and if they have endometriomas that are either bilateral or >4 cm in size. On the other hand, surgery is prioritized if the lesions are smaller, the ultrasound diagnosis is clear, the ovarian reserve is sufficient and spontaneous conception is still a reasonable goal (44).

Adverse effects of hyper-progestrogenic therapy (combined oral contraceptives and progestins) include sudden bleeding, nausea, breast tenderness, headaches and mood swings. Second-line drugs, which produce hypoestrogenic effects, can result in seborrhea, hypertrichosis, weight gain, migraines, hot flushes and a decreased libido, and can affect the lipid profile of the patient (30,45). The use of NSAIDs may be associated with adverse effects, including gastric ulcers, cardiovascular events and acute renal failure (30). In the study conducted by Lotz *et al* (46), it was found that premature ovarian insufficiency (POI) can occur in women without any known cause, but particularly following hormonal (gonadotoxic) therapies and can coexist with endometriosis. However, the treatment for POI is hormone replacement therapy, which can cause the recurrence of endometriosis and can cause bone loss (46). Treatment with a GnRH agonist can result in bone loss; the addition of estrogen or progesterone backup can prevent this effect during the therapy. The first-line treatment, oral contraceptives, offers contraception and is reasonably priced. However, medications such as dienogest and GnRH analogues are costly and lead to patient nonadherence (47). The Cochrane systematic review by Bafort *et al* (48) summarized the results of a comparison of adverse effects (vascular injury) of surgical techniques, such as ablation and nerve transection, against diagnostic laparoscopy; however, the available data were inadequate and inconclusive to determine the extent of vascular injury associated with each intervention (OR, 0.33; 95% CI, 0.01 to 8.32; one RCT, 141 participants; low-quality evidence) (48). Considering that endometriosis can affect quality of life in a variety of ways, including reproduction and mental health, and other effects. An alteration in the treatment method for endometriosis should be considered. It's necessary to highlight the flaws in the current standard of care, and new therapeutic approaches must be implemented (49).

## 4. Emerging medical therapies

Timely diagnosis and accurate treatment will improve the fertility concerns and the quality of life of the patient. A substantial minority (~1 third) of patients display progesterone resistance or an inadequate response to first-line hormonal therapy, which has driven interest in alternative and non-hormonal strategies. Laparoscopic exploratory and diagnostic surgery precisely identifies and removes endometrial tissue; however, 50% of the patients experience recurrence of lesions following these procedures. In patients who have undergone a hysterectomy, 25% experience recurrent pelvic pain. Nevertheless, the relief from these methods is temporary (50-52). A concise overview of the major emerging and established therapy classes, their mechanisms, reported development stage and principal safety considerations is provided in Table I.

### Oral GnRH antagonists (elagolix, relugolix and linzagolix)

Oral GnRH antagonists such as elagolix, relugolix and linzagolix were approved for management within the timeline of 2018-2025. Although these agents are already approved for clinical use, they are included in the present review as mechanism-driven therapies that represent a more recent generation of targeted treatments for endometriosis, in contrast to emerging strategies, such as epigenetic therapeutics and microbiome modulation, which remain at preclinical or early clinical stages of development. In 2022, the Food and Drug Administration (FDA) approved Myfembree^®^, a once-daily fixed-dose combination tablet containing 40 mg relugolix, 1 mg estradiol and 0.5 mg norethindrone acetate, for a 1-year course of treatment for moderate to severe endometriosis-related pain in premenopausal women (53,54). In a meta-analysis conducted in the year 2023, details about the efficacy and side-effects of these drugs were provided (55). Pelvic pain was considerably decreased, and the efficacy of the drugs was ranked: Elagolix 400 mg (P-score: 0.07), linzagolix 75 mg (P-score: 0.23), linzagolix 200 mg (P-score: 0.25), elagolix 150 mg (P-score: 0.54), linzagolix 50 mg (P-score: 0.55) and elagolix 250 mg (P-score: 0.60) (55). As regards safety, decreased bone mineral density (BMD) was one of the concerns associated with GnRH antagonist therapy. In a separate phase 3 randomized, double-blind, noninferiority study by Harada *et al*, BMD changes from baseline to the end of 24 weeks of treatment were -4.80% in the relugolix group and -4.84% in the leuprorelin group (56). Elagolix therapy was also connected to bone density loss. Results from a double-blind, randomized, placebo-controlled, parallel-group study by Carr *et al* (53), stated that the administration of high-dose add-back therapy (ABT) (1.0 mg estradiol/0.5 mg norethindrone acetate) significantly mitigated this effect. Hot flushes were often reported as an undesirable effect; however, the inclusion of ABT helped relieve this symptom as well (53).

### Selective progesterone receptor modulators (SPRMs)

SPRMs are synthetic compounds that target the progesterone receptor with both agonistic and antagonistic activity. Their therapeutic potential is based on their effect on progesterone signaling. The anti-progestogenic action of vilaprisan, a novel drug, is 5-fold greater than that of ulipristal acetate (57). As previously demonstrated, vilaprisan at 0.5-4 mg for up to 3 months or vilaprisan at 2 mg for up to 6 months was well-tolerated in a phase 1 trial involving healthy women and phase 2 and 3 trials involving women with uterine fibroids (57).

### Aromatase inhibitors (AIs)

Exemestane is a type 1 steroidal AI that can cause irreversible inactivation by directly competing with androgens for the active site of the enzyme. On the other hand, letrozole and anastrozole are the second type present in this group that bind reversibly to the aromatase by competing with androgens for the binding sites. In the systematic review conducted by Garson *et al* (58), the results of a single-arm open-label trial that included 10 premenopausal women who experienced refractory or recurring pelvic pain were summarized. Each patient had a diagnostic laparoscopy 1 month before commencing treatment with AIs and 3 months after terminating medication. Treatment involved 2.5 mg letrozole and 2.5 mg norethindrone acetate taken daily for a period of 6 months. At the end of the treatment period, after 1 month, a laparoscopy revealed a significant decrease in the ASRM score and endometriosis stage (44.1±29.7 against 5.4±5.64); 9 out of 10 women reported an improvement in symptoms and a marked reduction in pain (mean pain: 6.22±2.07 vs. 2.52±2.09; P<0.001) (58).

### Non-hormonal small molecules and other anti-inflammatory agents

According to the benign metastasis theory, endometriotic lesions are lymphatic/hematogenous disseminations of endometrial tissue. A previous study demonstrated that endometrial tissue was present in lymph nodes in 6-7% of lymphadenectomies. mTOR and Raptor expression were shown to be considerably higher in patients with endometriosis (P≤0.0001); Deptor expression was not altered, and Rictor expression was significantly higher in patients aged 20-29 years (P=0.0004) (59).

Pentoxifylline, a methylxanthine that functions as a phosphodiesterase inhibitor, has anti-inflammatory properties, and it has been suggested as a treatment for endometriosis. In the year 2021, Grammatis *et al* (60) stated that the subjects were monitored for a full year, while taking 800 mg pentoxifylline daily and found that there was no statistically significant difference in the overall and recurrent rates of pain complaints with the use of this drug.

Metformin was another drug that was found to be useful in the treatment of endometriosis. Its effect is mediated through the alterion of the production and activity of inflammatory mediators, such as IL-1β, IL-8 and PGE2. It also decreases the expression and secretion of Wnt2 protein in ectopic endometriotic stromal cells, which suggests the importance of future investigations on the effects of metformin on endometriosis (61).

### Antioxidants and nutraceuticals

Astaxanthin (dihydroxy-4,4'-dione-β,β'-carotene; AST), also known as the ‘king of antioxidants’, is a red-orange, lipid-soluble, xanthophyll photo-pigmented ketocarotenoid that was extracted from the algae *Haematococcus pluvialis*. The impact of AST on pro-inflammatory cytokines, oxidative stress markers, and early pregnancy outcomes was studied in a randomized, triple-blind, placebo-controlled clinical trial (RCT) by Rostami *et al* (62), involving 50 infertile women with endometriosis (at different stages) who were candidates for ART. Following treatment with AST, blood levels of TNF-α (2.520±0.525 vs. 2.968±0.548; P=0.038) and IL-1β (4.515±0.907 vs. 6.8760±0.8478; P=0.000) were significantly lower. Therefore, supplementation with AST may be a potential way to combat the inflammatory response and oxidative stress linked to endometriosis (62).

### Anti-angiogenic approaches and MMP modulation

An increase in pro-angiogenic factors is observed in patients with endometriosis as a result of angiogenesis. This causes the microvascular network to form and ectopic endometrial vessels to become neovascularized. anti-VEGF antibodies, VEGFR tyrosine kinase, COX-2 inhibitors and dopamine agonists are the primary angiogenic drugs used for enhancing patients' symptoms and being useful for clinical treatment (63). As previously demonstrated, a patient treated for severe endometriosis with bevacizumab (Avastin, a monoclonal antibody directed against VEGF) in a human clinical trial reported that her therapy-refractory dysmenorrhea disappeared completely. During the second-look laparoscopy, diffuse fibrosis of her endometriosis lesions was also noted. In endometriotic stromal cells, statins suppress inflammation and the angiogenic genes VEGF and cyclooxygenase-2. The dopamine agonist cabergoline was also attributed to be a useful treatment for endometriosis-related chronic pelvic pain and also in its prevention, potentially by modulating VEGF/VEGFR-2 signaling (23). In the randomized exploratory trial by Kodarahmiann *et al* (64), it was shown that in women with endometriosis, the expression of MMP-2 and MMP-9 was elevated in both the eutopic endometrium and endometriotic lesions. In the randomized exploratory study (n=17) by Kodarahmiann *et al* (64), the role of resveratrol in angiogenesis, apoptosis, proliferation and the production of oxygen radicals was established and it was found to be associated with the dramatically decreased levels of MMP-9 protein expression level both within the treatment group (P<0.001) and in comparison to the control group (P<0.001), which plays a crucial role in the ectopic implantation of endometrium. In the randomized exploratory trial by Kodarahmiann *et al* (64), resveratrol significantly reduced the MMP-9 concentrations in endometrial fluid (347.5±43.0 vs. 422.3±44.9 ng/ml) and serum (591.2±70.1 vs. 738.5±101.1 ng/ml) compared with the control group (P<0.001). Significant reductions in endometrial fluid MMP-9 levels were also observed within the treatment group following the intervention, whereas no significant changes were observed in the control group (64).

### Epigenetic therapeutics

Psilopatis *et al* (65) summarized the results from a study conducted by Xiaomeng *et al* (66) in which low levels of histone H4 acetylation in both eutopic and ectopic endometrial tissues were identified by tracking the histone modification profile of endometriosis patients. Additionally, there was a considerable downregulation of Sirtuin 1 mRNA in the eutopic endometrium, a marked decrease in histone deacetylase (HDAC)1 mRNA expression in the ectopic endometrium, and a significant upregulation of HDAC2 mRNA in th eutopic endometrial tissue. The role of regulatory and epigenetic pathways in endometriosis was also investigated. Several drugs such as N-butyrate, entinostat, vorinostat, valproate, and trichostatin-A, exhibit anti-endometriotic properties, which can be utilized in the future. A decrease in lesion size impairs cell survival and causes histone hyperacetylation. Apicidin and valproic acid, when used together, further inhibit proliferation, stop the cell cycle, cause apoptosis and downregulate Bcl-2/Bcl-xL. When taken in combination with progesterone or levo-tetrahydropalmatine, valproic acid exhibits additional advantages (66).

The pathophysiology of endometriosis is intricately linked to epigenetic instability. Changes in DNA methyltransferases, HDAC, histone methyltransferases and other enzymes that modify chromatin epigenetically to affect its structure and function are observed in both endometriotic lesions and the eutopic endometria of women with endometriosis. Preclinical testing of epigenetically targeted medications, such as HDAC inhibitors and HMT inhibitors, has already resulted from a basic understanding of these mechanisms and shows some promise (12). However, the translation of these epigenetic findings into clinical therapies remains in its early stages. No clinical trial has yet evaluated HDAC inhibitors in endometriosis, highlighting the need to establish their efficacy and safety. Moreover, as HDACs are ubiquitously expressed, systemic inhibition may lead to off-target effects, and localized delivery approaches, such as an HDACI-eluting intrauterine device, have been proposed to improve safety (66). Additionally, further studies are required to characterize the mechanistic associations between specific epigenetic modifications, lesion subtypes, disease stages and clinical outcomes to facilitate the development of targeted therapies (12).

### Microbiome modulation

Endometriosis is one of the disorders for which correcting gut microbiota imbalances and returning them to their normal functioning state has emerged as a novel therapeutic option (19). In the comparison study conducted by Ata *et al* (67), the differences in the composition of cervical, vaginal and gut microbiota were examined, and women with stage 3-4 endometriosis and control subjects appeared to have similar overall microbiome compositions in the cervix, vagina and gut; there appeared to be some genus-level variations (67). *Lactobacillus* supplementation and its beneficial effect on pain symptoms associated with endometriosis were summarized by Xholli *et al* (19) from a pilot RCT by Khodaverdi *et al* (68). LactoFem^®^, a formulation containing *Lactobacillus*, was administered orally once daily for 8 weeks to women with stage 3 and stage 4 endometriosis. The trial included 37 patients who had not received hormonal treatment in the previous three months. After using *Lactobacillus* for 8 weeks, the largest improvement in pain score was shown (1.09±1.00 vs. 1.34±0.06) (19,68). A retrospective study conducted in 2025 demonstrated that microecological therapy significantly improved intestinal mucosal immune function in patients with endometriosis (69). Treatment was associated with significantly increased levels of CD4^+^ T-cells (425.8±38.9 vs. 312.6±45.2 pg/ml; P<0.001) and secretory IgA (59.8±9.4 vs. 42.3±8.1 µg/ml; P<0.001). Conversely, inflammatory markers, including TNF-α (72.5±10.6 vs. 103.2±14.2 pg/ml; P<0.001), IL-6 (56.9±7.8 vs. 84.5±10.3 pg/ml; P<0.001) and CD8^+^ T-lymphocytes (297.6±41.7 vs. 398.3±52.4 pg/ml; P<0.001), were significantly reduced following treatment (69). However, the findings of that retrospective study should be interpreted with caution because of potential selection bias, variations in clinical practices, the use of a single synbiotic formulation, and limited follow-up. Larger, prospective randomized controlled trials with standardized protocols and longer-term follow-up are required to validate these findings and establish the clinical efficacy of microbiome-based therapy (69).

### Device-based and novel non-pharmacologic approaches

The efficacy and safety of a novel pain management device, AT-04, were studied in a phase III RCT. AT-04 is an ultralow-invasive device that can be used without preventing ovulation in women with endometriosis. As the mechanism of pain management of AT-04 differs significantly from that of current therapies, it may be effective in treating endometriosis-related pain in women (70).

Due to the uncertainty in the pathogenesis of the condition, which is currently attributed to metastasis theory, microbiome dysbiosis, immunologic mediators and aberrant signaling pathways such as the PI3K/AKT/mTOR pathway, further investigations on these agents are warranted to support the creation of innovative treatments (19,59,71).

## 5. Surgical/device innovations and procedural adjuncts

In recent years, the surgical management of endometriosis has moved well beyond standard laparoscopy toward a range of technical adjuncts and device-enhanced strategies aimed at improving excisional completeness, reducing morbidity and improving outcomes.

### Minimally invasive excisional techniques

Minimally invasive excisional technique refinements and device innovation, such as the evolution of laparoscopic image quality and retroperitoneal anatomical awareness, have pushed endometriosis surgery from blunt thermal ablation toward precise, anatomy-preserving excision and lesion-targeted adjuncts. There has been a shift from rigid stepwise protocols toward a more flexible, anatomy-based framework. Fleischer *et al* used a mnemonic SOSURE to describe a framework on a number of operative steps which will help in maximizing access and optimizing assistance during endometriosis surgery (72). Such surgical steps ensure a clear exposure of pelvic organs, which in turn provide great independence for the lead surgeon to operate/dissect (72). These include the following: S, survey/sigmoid mobilization; O, ovarian mobilization; S, suspension of the uterus and ovaries; U, ureterolysis; R, rectovaginal and pararectal spaces; E, excision of all endometriotic lesions (72).

### Robotics and vNOTES

Even though minimally invasive surgery remains the cornerstone of endometriosis management, robot-assisted laparoscopy has become particularly valuable for complex deep infiltrating endometriosis (DIE). Robotic assistance provides three-dimensional visualization and stabilized equipment, allowing for the detailed removal of endometriotic lesions and precise movements during critical anatomical dissection. Examples include the da Vinci Xi and da Vinci SP; these systems can enable intricate dissections with fewer technical difficulties and smaller incisions in difficult-to-reach areas, such as the rectovaginal septum or pelvic sidewalls (73).

Kanno *et al* (74) reported performing robot-assisted ureteroneocystostomy with hysterectomy using the da Vinci SP. The patient was a 49-year-old woman with uterine fibroids, left hydroureter and grade 4 hydronephrosis with ureteral endometriosis. The surgery was completed without any complications, and the post-operative course was also uneventful. Meticulous dissection, suturing and knot-tying have been challenges in conventional single-port laparoscopic surgery; the articulating instruments of the da Vinci SP may simplify these tasks. The robotic approach may be particularly useful in complex resections involving significant organ compromise (74).

Sinha *et al* (75) reviewed the role of robotic surgery in gynecology and noted that hybrid robot-assisted approaches have been reported for complex colorectal endometriosis. In their discussion, they cite a case series in which both conventional laparoscopy and the da Vinci system were used to treat 7 infertile women with severe bowel disease; all achieved disease-free margins, all reported symptomatic improvement at 3, 6 and 12 months, and 2 patients >35 years of age conceived spontaneously. Overall, Sinha *et al* (75) concluded that robot-assisted laparoscopy appears to be a feasible option for achieving comprehensive surgical management of endometriosis.

The full potential of robotic surgery is utilized in robot-assisted transvaginal NOTES (RvNOTES) to enhance patient outcomes. It has been observed that surgeries using RvNOTES reported reductions in pelvic pain associated with endometriosis (73).

### Intraoperative imaging [indocyanine green (ICG)/near-infrared NIR)]

ICG is currently used extensively in gynecological surgical procedures for identification of lymphatic tissue. A major adjunctive innovation is the use of fluorescence imaging with ICG and NIR systems to improve intraoperative lesion detection, margin delineation and perfusion assessment (76). As previously demonstrated, the operative field was first observed and classified under white light and then injected with ICG and observed and classified under NIR. Using both techniques, 240 suspicious lesions were found. Out of the 240 lesions, 207 (86.2%) were visible with white light imaging, and 200 of those lesions were verified to be pathogenic (true-positive for white light). Only NIR-ICG imaging was able to detect the remaining 33/240 (13.75%) (false-negative for white light) lesions, and all 33 of the excised lesions were verified to be pathogenic. NIR-ICG vision demonstrated a positive predictive value of 98.5%, a negative predictive value of 87.1%, sensitivity of 87%, and a specificity of 98.5%, indicating that this type of imaging is a good diagnostic and screening test (P=0.001 and P=0.835, respectively according to McNemar's and Cohen's kappa tests) (76). Therefore, it is a valuable tool for discriminating against diseases from healthy tissue.

Moreover, recent research suggests ICG imaging is feasible in endometriosis surgery for bowel/ureter/perfusion assessment. Raimondo *et al* (77) examined uterine perfusion using ICG imaging, and critical intraoperative decisions, such as stent insertion, were simplified by its exceptional ability to detect ischemic areas. Following conservative ureteral surgeries, they examined 31 ureters using NIR-ICG imaging. The mean assessment time was 5.4±2.3 min. Under normal white-light inspection, surgeons suspected poor blood supply (ischemia) in 5 ureters. Following the addition of ICG, 2 of those 5 ureters (40%) exhibited normal fluorescence; thus, they did not need a stent. The other 3 ureters (60%) exhibited weak or no fluorescence, confirming poor perfusion; thus, a stent was placed (77).

### Nerve-sparing approaches

Contemporary methods, such as the nerve-sparing approach for the treatment of DIE is typically used for the identifying and preserving the integrity of the nervous system of the affected organ. Nerve-sparing surgery effectively reduces the incidence of bowel, bladder and sexual dysfunction, while maintaining surgical efficacy (78). Ianieri *et al* (79) reported a single-center, observational, retrospective study on symptomatic women who underwent the laparoscopic excision of deep-infiltrating endometriosis (DE) with segmental bowel resection using a nerve-sparing technique and with inferior mesenteric artery preservation between September, 2019 and April, 2022. A total of 61 patients were enrolled in which intraoperative complications were rare, and no major (Clavien-Dindo grade III-IV) post-operative complications occurred. None of the patients experienced rectovaginal, vesicovaginal, ureteral or vesical fistula, or ureteral stenosis. There was also no report of reintervention. Crucially, gastrointestinal symptoms significantly improved after 6 months according to validated functional ratings (Knowles-Eccersley-Scott-Symptom and Gastro-Intestinal Quality of Life Index questionnaires), supporting the notion that nerve-guided conservative resection can preserve organ, while effectively clearing the disease. In total, 5 patients (8.3%) experienced bladder voiding deficits, and 6 patients (9.8%) had urinary tract infections. Low rates of intraoperative and postoperative complications were achieved by preserving the main vascular arch of the rectum and performing a nerve-sparing dissection of the mesorectal and parametrial planes. In particular, anastomosis leakage occurred in only 1.6% of cases (1 patient who had previously undergone surgery for DE) (79).

### Adhesion prophylaxis

Anti-adhesion barriers are adjuncts used during the surgical treatment of endometriosis to reduce post-operative peritoneal and pelvic adhesions that contribute to pain and infertility. In addition to placing a heavy financial strain on healthcare systems, peritoneal adhesions can cause patients enormous suffering, primarily in the form of pain, obstruction and secondary infertility. When a surgery is inevitable, adhesion prophylaxis should be strategized including improved surgical techniques, as well as adhesion prophylactic agents and devices. The use of adhesion barriers has yielded significant success in the field of gynecological surgery. Some types of barrier agents used are oxidized regenerated cellulose (ORC), polyethylene glycol (PEG), hyaluronic acid and hyaluronate (HA) + carboxymethylcellulose (CMC), icodextrin, dextran, fibrin-containing agents etc. (80).

Exogenous hyaluronic acid is a helpful anti-adhesive medication used in clinical settings to help patients avoid adhesions during gynecological procedures, such as laparoscopic endometriosis surgery (81). Hyaluronic acid, a linear polysaccharide composed of repeating sodium D-glucuronate and N-acetyl-D-glucosamine units, is an essential component of numerous bodily tissues and fluids, providing protection and physical support. It reduces inflammatory reactions and promotes wound healing in addition to acting as a mechanical barrier that promotes fibrin disintegration and mesothelial cell development. Therefore, hyaluronic acid aid in lowering the development of post-surgical adhesions during tissue regeneration (82). According to a previous meta-analysis of 12 clinical trials, patients receiving HA exhibited a significantly decreased percentage of adhesion-affected areas (P<0.00001) compared to the controls and a noticeably lower rate of moderate or severe adhesions (P=0.0010) (82). These results demonstrate the clinical utility of HA as an anti-adhesion barrier and encourage its wider use in reproductive procedures (82). Nevertheless, research on the long-term consequences, effectiveness and possible cytotoxicity or adverse effects of these implanted barriers remains insufficient, and there is no reliable evidence of their high efficacy in avoiding adhesions.

Innovations in endometriosis surgery and devices are becoming more advanced and promising. The field is being led by robotic-assisted laparoscopy, improved visualization/adjunct devices, and procedural improvements for organ and nerve preservation. In a number of areas, however, the evidence remains exploratory; standardized comparison trials, cost/implementation studies and long-term outcome data are required.

## 6. Biomarkers, diagnostics and precision medicine

Endometriosis remains markedly underdiagnosed due to long delays in diagnoses; non-invasive biomarkers are therefore highly desirable to move care from empiricism toward precision. Despite hundreds of candidate markers (e.g., CA-125, CA-19-9, IL-6 and urocortin) measured in blood, urine and the endometrium, no clinically validated non-invasive biomarker or assay has yet emerged. Study heterogeneity, small cohorts, variable sample types and poor reproducibility are the main barriers (83,84). A summary of candidate diagnostic biomarkers, their current validation status and principal limitations is provided in Table II.

### Current clinical imaging (mature biomarkers)

Imaging biomarkers are clinically mature: Transvaginal ultrasound and MRI are now first-line for diagnosis and pre-surgical mapping, with MRI providing the multi-compartment assessment of deep pelvic endometriosis involving the uterus and its ligaments, adnexa, bowel, distal ureters, urinary bladder and pelvic nerves. Additionally, MRI can help detect extra-pelvic organ involvement as well. The use of a dedicated MRI protocol and structured reporting template improves multidisciplinary communication and provides a pre-surgical road map; it also aids in patient counseling, as well as in assessing the need for additional intraoperative organ-specific expertise, such as colorectal surgery or urology (85).

Combining the anatomical detail of MRI with the functional/molecular information from positron emission tomography (PET) could, in principle, yield a more comprehensive road map; for surgeons, particularly for complex or multi-site disease. However, when Oldan *et al* (86). conducted a single-site, prospective pilot study on 6 patients by administering 18F-fluoroestradiol and then imaging them using a Siemens mMR PET-MR scanner, PET did not prove to be as sensitive as MR (50 per-patient sensitivity vs. 67% per-patient and 35 vs. 48% per-lesion) and did not show any additional sites over and above MR. They did not find that the addition of PET imaging (with or without comparison with MRI) provided greater diagnostic benefit over MRI (86).

Despite these limitations, the concept remains attractive, as PET enables molecular-level imaging which could allow non-invasive detection of lesions that are otherwise occult on conventional imaging.

### Failure of single blood/urine marker

According to Tian *et al* (84), CA-125 levels vary with the menstrual cycle, disease type and stage, and IL-6 has a limited diagnostic ability on its own, even though it has been evaluated in multiple studies. As mentioned in the review Tian *et al* (84), several authors therefore recommend focusing on the efficacy of IL-6 combined with other cytokines rather than as a single marker. These reflect on the limits of the biomarkers and the generalizability of the test for a diagnosis (84).

### Metabolomics: Promise and metabolite signatures

Metabolomics is being investigated as a minimally invasive strategy with which to characterize endometriosis-related biochemical changes and to support early diagnostic development. However, existing studies still contain flaws and confounding variables. Several metabolite classes, particularly amino acids, lipids and organic acids, show consistent alterations (87). A systematic review by Brulport *et al* (88) identified 1,107 possible indicators in all nine biological compartments under study, indicating that endometriosis may be linked to extensive molecular alterations,yet many are likely false positives or technically driven (88).

Multiple analyses across serum, urine, follicular fluid, endometrial fluid and tissue have reported disrupted amino-acid profiles in affected patients compared with healthy controls. These findings are essential for elucidating some aspects of disease progression. The altered levels of amino acids potentially explain tissue injury repair mechanisms in endometriosis and the increased energy demand of proliferative endometrial cells. The roles of the majority of lipids reported by researchers are related to endometrial cell pathophysiology. Characteristic lipid profiles have been identified in different biological compartments. Increased levels of lysophosphatidylethanolamine, omega-3 arachidonic acid and phosphatidic acid, and decreased levels of phosphatidylcholine and phosphatidylserine have been observed in the eutopic endometrium (87). Organic acidsm, such as pyruvate, succinate and citrate are key intermediates of the TCA cycle, and some have been reported to be elevated in serum and follicular fluid (87).

### Most reproducible molecular candidates

Among the numerous biomarker candidates proposed for endometriosis, only four reproducible markers (TNF-α, MMP-9, TIMP-1 and miR-451) have been consistently detected across multiple biological compartments and in well-powered cohorts (88).

Of these, MMP-9 (enzyme matrix metalloproteinase) exhibits the most consistent increase across all compartments and is unaffected by cycle phase or fertility status. Huang *et al* (88) analyzed the data from multiple studies to assess the diagnostic value of serum MMP-9 concentration for endometriosis. A meta-analysis of 15 studies (n=996 cases, 582 controls) found a pooled SMD=1.31 (95% CI, 1.02-1.60), but with high heterogeneity (I² >80%), indicating promise but need for higher-quality confirmatory studies (89).

TIMP-1 demonstrates conflicting patterns (reduced in blood and ovarian tissue but increased in peritoneal fluid) and appears influenced mainly by menstrual cycle rather than disease severity. miR-451 performs well as a diagnostic marker across different biological fluids and study settings, especially in combination panels, with other miRNAs, though inconsistencies in direction of change indicate a need for better-controlled studies (88).

### Circulating cell-free DNA (cirDNA)

cirDNA is a source of emerging diagnostic and prognostic biomarkers relevant in oncology and other disease areas. When changes in cirDNA were found to be associated with common benign conditions, it was considered as an opportunity for a biomarker development for diseases such as endometriosis.

However, studies proved otherwise (90). Alonso *et al* (91) conducted a prospective study involving 28 women with endometriosis and 15 controls. Women with and without endometriosis had cirDNA concentrations of 2.24±0.89 and 2.56±0.92 ng/ml, respectively, with no significant differences observed between the two groups. Although women with superficial endometriosis exhibited significantly higher endometrial cirDNA concentrations than those with deep infiltrating disease (mean difference, 0.14 ng/ml; 95% CI, 0.15-0.26; P=0.025), cirDNA levels did not differ significantly between patients and controls (91). These findings suggest that cirDNA currently lacks sufficient evidence to support its use as a reliable standalone diagnostic biomarker for endometriosis. Nevertheless, given the limited sample size and heterogeneous control population of that study, further large-scale, well-designed studies are required to determine whether specific cirDNA subtypes or combined biomarker panels may have future diagnostic value (91).

### Translational and regulatory implications [companion diagnostics (CDx)/EU in vitro diagnostic regulation (IVDR)]

The EU currently treats CDx as a distinct regulatory category under the IVDR, requiring CE-certification, robust performance/clinical evidence and notified-body oversight; biomarkers intended as CDx must comply. As CDx use was inconsistently recorded before the IVDR, a number of trials lack validated companion assays. In order to collect information on the use of CDx testing in clinical trials, a thorough database search technique was created. A strategic assessment for possible CDx designation under IVDR was made possible by the analysis of trials based on medicinal products, biomarkers, and *in vitro* diagnostics. This analysis also helped with the evaluation of medicinal products and related IVD tests (92).

## 7. Clinical trial landscape, regulatory status and approval pathways

A marked rise in endometriosis trials has been observed in recent years, reflecting a shift beyond conventional therapies. According to He *et al* (93), 744 trials were registered across registries as of April, 2025. Of the 387 active trials on ClinicalTrials.gov, 116 (~30%) dealt with interdisciplinary or novel methodologies (94).

Early phase trials (phase I-II) are increasing, particularly oral GnRH antagonists, such as elagolix, relugolix and linzagolix, which have exhibited the most success (93). For instance, the results from the Elaris EM-I trial (n=872), indicated that dysmenorrhea responder rates were 46.4% with 150 mg daily elagolix and 75.8% with 400 mg daily, compared to 19.6% with placebo. A similar result was observed in Elaris EM-II (n=817): 43.4 and 72.4%, compared with 22.7% with the placebo (95).

Elagolix, the first GnRH approved by the US FDA in 2018 for moderate to severe endometriosis pain, has exhibited dose-dependent efficacy in key trials: Daily doses of 150 and 400 mg reduced dysmenorrhea and non-menstrual pelvic pain (NMPP) vs. the placebo; however, higher doses were linked to increased hypoestrogenic effects and notable BMD loss (up to -3.9% at 12 months) (96). A 48-month phase III trial (NCT03213457) is evaluating elagolix combined with ABT; findings revealed improvement in dysmenorrhea, NMPP and fatigue (93).

Another approved GnRH, relugolix (US FDA, EMA), also exhibited potential when administered as combination therapy. the phase III SPIRIT 1 trial (n=638) reported responder rates for dysmenorrhea of 75 vs. 27% for the placebo and NMPP of 59 vs. 40% for the placebo; in the phase III SPIRIT 2 trial (n=623), the respective dysmenorrhea rates were 75 and 30%, and NMPP rates 66 and 43% for the placebo, demonstrating an improvement in dysmenorrhea and NMPP with a minimal decline in BMD (97).

Similarly, linzagolix with ABT in the phase 3 EDELWEISS-3 trial (n=486) reported 72.9% dysmenorrhea responders on linzagolix 200 mg + ABT vs. 23.5% on the placebo, and 47.3 vs. 30.9% for NMPP (98). These trials demonstrated that GnRH antagonists can be effective for endometriosis; however, systemic effects, particularly on BMD, need to be managed appropriately.

Several non hormonal therapies are under evaluation. Early development of SPRMs such as vilaprisan showed efficacy signals, but the programme was terminated on safety grounds (57,98). P2X3 receptor antagonists (eliapixant and gefapixant), an emerging non-hormonal class, were explored endometriosis-related pain, but recent trials have been disappointing, showing no clinical improvement (93).

As endometriosis has a pro-inflammatory milieu, immune-targeted therapies, such as TNF-α inhibitors have been investigated. However, clinical data to date have not shown a meaningful benefit for infliximab (99,100). These results highlight the complexity of endometriosis pain mechanisms and the challenges of finding effective non-hormonal therapies. Key randomized and pivotal trials, their designs, headline outcomes and follow-up intervals are summarized in Table III.

Regulatory pathways have expanded alongside clinical research. Elagolix provides an advantage over conventional therapies, such as oral daily dosing and a short half-life that allows for rapid symptom relief with a more rapid hormonal recovery (92,101). Other oral GnRH have followed, such as the combination of relugolix with ABT in some regions (under the name Ryeqo in Europe, initially for uterine fibroid symptoms) and is under review for endometriosis related pain (101). In 2022, the FDA approved a similar relugolix combination (under the brand name Myfembree) for endometriosis, expanding GnRH antagonists for endometriosis in the USA (102,103). However, regional differences in approvals and access remain. For example, the relugolix combination (Ryeqo/Myfembree) has approvals in the EU and USA, whereas elagolix is primarily available in the USA and has not been submitted for EU marketing authorization (104). Besides oral GnRH antagonists, no other novel therapies have achieved regulatory approval for endometriosis to date. Overall, the regulatory landscape remains cautious and varied across regions. New treatments must demonstrate both acceptable long-term safety and effectiveness in reducing pain.

The design of endometriosis clinical trials has faced challenges with critical implications for interpreting the emerging therapies. The main goal of the majority of trials has been to reduce pain, usually assessed pain measures for dysmenorrhea and non-menstrual pelvic pain (93,96-98,105,106). Improvements in quality of life (QoL) is a key secondary endpoint often measured with tools, such as Endometriosis Health Profile-30 (EHP-30) (97,107). EHP-30 is a patient outcomes measure acknowledged for its clinical value in evaluating the complex effects of endometriosis across domains such as pain, control, powerlessness, social support, emotional well-being and self-image. Since it is a disease-specific questionnaire that is intended to capture the distinctive and complex aspects of the condition, it is the recommended tool for endometriosis research (107).

Even though QoL is a crucial secondary goal, standardized reproductive outcomes, such as viable intrauterine pregnancy, live birth and time to pregnancy should be collected when needed. This suggestion emphasizes the underreporting of fertility endpoints in studies (106). This leaves a gap about how novel treatments affect fertility, a critical unmet need for patients desiring future pregnancy. Another limitation is the short follow-up duration in the majority of trials (typically 3-6 months), as observed in pivotal phase III trials, such as the SPIRIT I (NCT03204318), SPIRIT II (NCT03204331) and EDELWEISS-3 (NCT03992846) trial. Such follow-up may suffice to show pain reduction but is insufficient to evaluate long-term outcomes, recurrence after treatment cessation, or sustained safety (97,98). Longer extension trials exist for some therapies (for example the 2-year SPIRIT open-label extension); however, long-term data on recurrence and chronic safety are limited for most novel therapies (105).

Efforts are underway to standardize and improve trial design in response to these challenges. An international consortium has developed a core outcome set for endometriosis trials to ensure studies report a minimum, consistent set of endpoints (106). The consortium recommends that trials should include at least a few of the following: pain symptoms, QoL, improvement in the most troublesome symptom, fertility/pregnancy, adverse outcomes and patient satisfaction with treatment (106). By adopting these standardized endpoints, future trials will be more comparable and the evidence base for novel therapies strengthened.

## 8. Safety, tolerability, fertility and contraceptive considerations

The FDA recently approved elagolix for the treatment of moderate to severe pain associated with endometriosis. Its effectiveness was demonstrated following 6 months of treatment; however, the primary adverse effect was a marked reduction in BMD, along with side-effects related to hypoestrogenism, such as hot flashes, dry vagina, nausea and headaches (108). As a supplement to GnRHa medication, ABT, which involves taking a small amount of hormone every day, is frequently recommended to reduce these side-effects. According to the website of the manufacturer, AbbVie, adverse symptoms usually resolve within 3 to 6 months after treatment is stopped (45). According to the European Society of Human Reproduction and Embryology (ESHRE) guidelines (2022), GnRH agonists are recommended to prevent bone loss and hypoestrogenic symptoms when combined with hormone ABT; GnRH antagonists are also used as second-line treatment, although evidence about optimal dose and duration is limited (109).

Elagolix at 200 mg twice daily has been linked to hypoestrogenic effects, such as decreased BMD, in phase-3 studies of women with endometriosis-related pain; this dose is therefore permitted for a maximum of 6 months. The hypoestrogenic effects of elagolix, such as BMD loss, can be mitigated by hormonal ABT (1 mg estradiol/0.5 mg norethindrone acetate once daily). ELA 300 mg twice daily plus ABT once daily has been approved in the USA for heavy menstrual bleeding linked to uterine fibroids in premenopausal women (110).

It has been shown that the risk of developing thrombosis is high in women with endometriosis; however, the mechanisms involved remain unclear. It is clearly evident that the coagulatory system in the human body is deeply linked to inflammatory responses, thereby increasing the risk of thrombosis, along with other risk factors, including a sedentary lifestyle and hormonal imbalances (111). MYFEMBREE^®^ (relugolix, estradiol and norethindrone acetate) is contraindicated in patients with thrombotic or thromboembolic disease and in women who are smokers (103).

Women with endometriosis also exhibit immune dysregulation, with several mechanisms resembling those described in cancer biology. Immunosuppressive, cells such as myeloid-derived suppressor cells (MDSCs), regulatory T cells (Tregs), and regulatory B cells (Bregs) have been found in endometriosis patients. These cells not only impede a healthy immune response but also accelerate the course of the illness (112). Several studies suggest atypical endometriosis may represent a transitional stage between benign and malignant disease (113).

### Fertility and reproductive planning

Endometriosis does not cause infertility in all affected women; similarly, the absence of endometriosis does not guarantee fertility. Major pelvic diseases, such as adhesions and chronic intraperitoneal inflammation, likely contribute to reduced fertility in many affected women (114). Pregnancy outcomes in women with endometriosis are variable and may include higher risks of miscarriage, ectopic pregnancy, preeclampsia and other complications (115). Referral to clinics that combine surgical and IVF expertise and use a multidisciplinary approach is recommended for endometriosis-related infertility (11). Patient-doctor communication guidelines developed for other chronic illnesses or trauma are applicable and beneficial for patient-centered counselling in endometriosis (116).

## 9. Cost, accessibility and health system considerations

Endometriosis imposes a substantial economic burden in the USA, driven by lost productivity and a reduced quality of life. Early estimates indicated that, by 2002, the condition was responsible for approximately $22 billion in annual healthcare expenditures, with $17.3 billion attributed to direct medical costs and $4.7 billion to indirect losses stemming from diminished work output and earnings (117). However, only a small number of comprehensive systematic reviews of the economic burden have been published since 2006, leaving a gap in contemporary cost estimates (118). A systematic review of 47 studies reported a 12.9% prevalence of endometriosis among women undergoing laparoscopy in Middle Eastern cohorts (119). Access to reliable information on endometriosis remains limited in some countries, including Lebanon, and this deficit directly contributes to inadequate research activity, poor public and professional awareness, and delayed or missed diagnosis (120). Gynecologists should consider fertility preservation when assessing women who have endometriosis. The evaluation of ovarian reserve, the potential impact of surgery on fertility, and the choices for fertility preservation are important factors to take into account (121). The management of endometriosis-associated infertility requires a multidisciplinary, individualized approach tailored to disease subtype, severity and patient priorities (11).

## 10. Evidence gaps, limitations and quality of existing evidence

Endometriosis likely does not arise from one uniform biological pathway. The condition varies widely in its presentation, including the type of lesions, their location, the symptoms they produce, and how patients respond to treatment. This makes a single unifying explanation for its development improbable. This heterogeneity supports the view that endometriosis consists of multiple subtypes that follow different disease pathways shaped by distinct genetic and epigenetic factors and hence complicates biomarker discovery, clinical phenotyping and the interpretation of treatment effects (122).

As endometriosis and infertility are tightly linked in clinical practice, estimates of reproductive prognosis need to be reported alongside therapeutic outcomes (114). Serum AMH is a validated marker of ovarian reserve (follicle quantity); however, it is not a direct measure of oocyte quality or pregnancy potential. Consequently, reductions in AMH levels, as for example, following the laparoscopic treatment of ovarian endometriomas, should be interpreted with caution and not equated with definitive reductions in fertility without accompanying pregnancy or live-birth data (123).

Population diversity is poor. Systematic reviews highlight predominantly Caucasian cohorts and paucity of data from racial/ethnic minorities, limiting generalizability and perpetuating health disparities (6). The lack of diversity in the research related to endometriosis can be bridged by building national and international connections between healthcare professionals, researchers, and patients (124).

Reporting deficiencies such as incomplete demographic data, selective outcome reporting and likely publication bias, further distort the evidence base. The Core Outcomes in Women's and Newborn Health (CROWN) initiative and its adoption in trial design represent important steps to standardize endpoints and reduce selective reporting. Conclusions are given based on randomized trial reports from these outcomes, and a reasonable explanation is given for the core outcomes that have not been collected (106).

It is clearly necessary to have more detailed studies and trials to obtain a better understanding of the disease, to achieve timely, proper, and accurate diagnosis, and to improve long-term QoL (109). Furthermore, to fully understand the factors contributing to the disease and ensure that clinical and biomedical advancements benefit everyone, not merely specific groups, research studies need to take into account and involve diverse populations, including those from racial and ethnic minorities and transgender individuals (125).

## 11. Future directions

Current analyses of the global endometriosis trial landscape indicate several key priorities for advancing treatment in the coming years (93). Recent expert recommendations stress the need for large, inclusive, multicenter cohorts with harmonized biobanking and core outcome sets to enable robust discovery, analytic validation and equitable deployment of non-invasive biomarkers and therapies (124).

### Conducting high-quality trials with fertility outcomes and long-term follow-up periods

Clinical trials must assess short-term pain relief and fertility outcomes (pregnancy, live birth) and adverse effects (106). Future studies should include women desiring pregnancy or follow patients after treatment to monitor natural conception rates; endpoints, such as time-to-pregnancy and live birth will address a major unmet need in endometriosis care (106). Longer follow-up is crucial to meet core outcome recommendations, track pregnancy after therapy, evaluate sustained pain relief, capture adverse effects, measure patient satisfaction, and determine long-term safety (96,99,106).

Trials should prespecify diversity and inclusion targets, link participants to registries and biobanks, and incorporate subgroup analyses so results are generalizable across populations and relevant to health equity (124).

### Biomarker driven patient stratification

Trials should embed biomarker research to enable precision medicine and patient stratification. Stratifying participants by validated biomarkers could increase trial efficiency and reduce failure rates by matching therapies to disease biology (93,124).

### Combination therapies

Combination regimens may outperform monotherapy given the multifactorial biology of endometriosis. Examples already in practice include GnRH antagonists with ABT (e.g., relugolix + estradiol/norethindrone acetate) to mitigate hypoestrogenic effects (105). Combining immunomodulators with hormonal agents or ABT may enhance efficacy, while reducing adverse events (126). Evidence for a number of non-hormonal agents (e.g., cabergoline, cannabinoid-based therapies) remains preliminary and requires controlled trials to confirm safety and efficacy. However, these agents, when considered alongside anti-inflammatory, antioxidant, microbiota modulating, or neuromodulatory therapies, may serve as a framework for tailored regimens that target the various biological mechanisms of endometriosis (126).

### Device and local drug delivery trials

The goal of new localized delivery methods is to lower systemic adverse effects and boost efficacy. For example, devices that locally release agents, such as gestrinone, an antiestrogen/antiprogesterone are under investigation to reduce pelvic pain with fewer systemic adverse effects (127).

### Safety registries and post-marketing surveillance

As novel therapies emerge, establishing safety registries and robust post marketing surveillance is crucial to monitor adverse effects and outcomes (95). Endometriosis typically requires long durations of treatment; thus, it is crucial for the systematic collection of safety data to monitor any adverse effects and outcomes (such as BMD) (96,99,106).

### Implementation science and cost effectiveness studies

Even highly effective treatments may fail to benefit patients if they are not accessible or implementable. High costs of novel therapies raise equity concerns. Implementation research and cost-effectiveness analyses are needed to guide policy and equitable access (96). Implementation studies should explicitly evaluate affordability, health-system feasibility and equity impacts to avoid exacerbating disparities in access to novel therapies (124).

In the coming years, results from current ongoing trials will determine future practice. Phase-3 readouts, such as the linzagolix EDELWEISS-3 trial, demonstrate clinically significant decreases in dysmenorrhea and NMPP, and serve as the foundation for any future regulatory applications. Long term safety, response durability, and the extent of clinical adoption will be enhanced by further late phase studies, longer follow up periods, and real-world data (98). Furthermore, there is an increasing emphasis on standardized outcome reporting, such as the pain numeric rating scale (NRS), QoL indices, fertility, recurrence rates and adverse effects that should be routinely reported to enable cross-study comparisons and improve patient outcomes and satisfaction (106). Recent trial findings also highlight unmet needs: Expanding access to novel medical treatments, assessing cost-effectiveness, obtaining real-world safety data and ensuring individualized care (for example, targeting the most troublesome symptoms of patients). These priorities reinforce that future studies should extend evidence for efficacy and safety and enhance implementation, access, and shared decision-making (98). To address current research gaps, increase awareness, and enhance the lives of patients everywhere, international collaboration amongst researchers that particularly support inclusive precision medicine research in endometriosis is crucial.

## 12. Conclusion

In summary, a number of treatment approaches based on mechanisms such as abnormal sex-steroid signaling, epigenetic changes, chronic inflammation, immunological dysregulation, angiogenesis and neurogenic sensitization have been made possible by recent advances in endometriosis biology. These discoveries have contributed to the development of targeted methods that go beyond managing symptoms. Prominent medical approaches discussed include refined hormonal modulators (oral GnRH antagonists, SPRMs and aromatase inhibitors) and a range of non-hormonal options, such as immunomodulatory biologics, anti-inflammatory and metabolic agents, antioxidants and anti-angiogenic compounds. Probiotics, antibiotics and dietary changes are examples of nutraceutical and microbiome-directed therapies that are being researched to reduce oxidative stress, inflammation and hormone imbalance. Although a number of these treatments remain in the early stages of clinical development and require further approval, taken as a whole, these demonstrate a move toward individualized, mechanism-based care.

Surgical and device innovations complement pharmacological advances. Improved minimally invasive and robotic procedures, increased intraoperative imaging (for example, indocyanine green fluorescence), nerve-sparing excision and adhesion-prevention tactics aim to provide more thorough, precise eradication of illness while conserving anatomy and fertility. Early evidence suggests potential benefits for pain and reproductive outcomes, but robust comparative trials and longer follow-up are lacking. For patients who are unable or unwilling to employ medicinal therapy, new ultralow-invasive pain-control devices may provide non-hormonal options.

Diagnostic and precision-medicine gaps continue. Standard imaging (transvaginal ultrasound, MRI) remains beneficial for mapping, whereas improved functional imaging has not proven significant increased benefit. Extensive biomarker research (proteomic, metabolomic, genomic) has identified candidates. For example, MMP-9 and certain microRNAs, but none are validated for routine clinical use. The field therefore requires reliable non-invasive biomarkers and companion diagnostics, along with standardized outcome measures (pain, quality of life, fertility endpoints) to strengthen future evidence.

To translate promise into practice will require large, well-designed trials with comprehensive endpoints, biomarker-guided and combination strategies, and attention to safety, fertility impact, cost, accessibility and equity. Coordinated, patient-centered research is essential to realize personalized, durable therapies for individuals with endometriosis.

## Figures and Tables

**Table I:** Therapies at a glance.

Therapy class	Example agents	Mechanism/target	Development stage	Key efficacy signal	Main safety/limitation	(Refs.)
Oral GnRH antagonists	Elagolix; relugolix (combo Myfembree); linzagolix	GnRH receptor antagonism → dose-dependent suppression of ovarian estrogen	Approved (elagolix FDA 2018); relugolix combo (approved 2022, FDA, EMA); linzagolix Phase-3 EDELWEISS-3)	Reductions in dysmenorrhea and non-menstrual pelvic pain; high responder rates reported in pivotal trials	Hypoestrogenic effects including BMD loss; mitigated by add-back therapy in some regimens	(53-56,95-98,107)
Selective progesterone receptor modulators (SPRMs)	Vilaprisan	Mixed agonist/ antagonist activity at progesterone receptor	Phase IIb (clinical development discontinued)	Early efficacy signals in non-endometriosis indications cited; limited endometriosis-specific trial data	Safety concerns led to termination of some development programs	(57,125)
Aromatase inhibitors (AIs)	Letrozole; exemestane	Block intra-lesional estrogen synthesis (aromatase inhibition)	Small clinical evaluations/ off-label uses reported	Single-arm trial reported decreased ASRM score and pain after letrozole + norethindrone acetate	Fertility implications; small sample sizes; need for add-back or progestin	(58)
Non-hormonal small molecules/ anti-inflammatories	Pentoxifylline; metformin	Anti-inflammatory (PDE inhibition for pentoxifylline); meta-bolic/anti-inflammatory effects for metformin	Clinical studies with mixed evidence	Pentoxifylline: no significant benefit reported in 1-year follow up; metformin: mechanistic rationale described	Inconsistent clinical efficacy; more robust RCTs required	(59-61)
Antioxidants & nutraceuticals	Astaxanthin (AST)	Antioxidant: Reduces ROS and inflammatory mediators	Randomized triple-blind RCT (n=50)	Reduced serum TNF-α and IL-1β after AST vs. placebo	Small trial sizes; fertility-outcome data limited	(62)
Anti-angiogenic and MMP modulation	Bevacizumab; cabergoline; statins; resveratrol	Target VEGF/angiogenesis pathways and MMP activity	Case reports, small trials and preclinical models cited	Case report: symptom resolution with bevacizumab; resveratrol reduced MMP-9 in small exploratory study	Systemic anti-angiogenics raise safety concerns; evidence preliminary	(23,63,64)
Epigenetic therapeutics	HDAC inhibitors (valproic acid, apicidin, vorinostat, entinostat); DNMT inhibitors (preclinical examples)	Reverse pathological epigenetic marks (HDAC/DNMT inhibition)	Preclinical and early translational studies	Preclinical lesion reduction; apoptosis and synergy with progesterone in models	Early stage; translational and safety data limited	(12,65)
Microbiome-targeted therapies	LactoFem^®^ (*Lactobacillus* formulation)	Restore estrobolome balance and mucosal immunity	Pilot RCT(s) and retrospective/early clinical studies reported	Pilot RCT (n=37) reported reductions in dysmenorrhea/ pain; 2025 retrospective microecological therapy study showed improved mucosal immunity markers	Small samples, heterogeneous study designs and methodologies, limited reproducibility and standardization across studies, and short follow-up; larger well-designed clinical trials with standardized protocols are needed.	(19,68,69)
Device-based/neuro-modulation	AT-04 ultralow-invasive device	Device-based pain management without ovulation suppression	Phase III RCT	Potential non-hormonal pain control option per phase III report	Mechanism distinct; requires replication and long-term outcome data	(70)
Surgical adjuncts and adhesion barriers	Robotic-assisted excision; ICG/NIR imaging; Hyaluronic acid barriers	Improve lesion detection, precision excision, and reduce adhesions	Clinical series/prospective studies/meta-analyses	ICG/NIR detected additional lesions not seen on white light (Gre-Endo trial); HA reduced adhesion area (meta-analysis)	Need standardized comparative trials and long-term outcome data	(72,73,75,76,81)

ART, assisted reproductive technology; ASRM, American Society for Reproductive Medicine; CPP, chronic pelvic pain; DIE, deep infiltrating endometriosis; GnRH, gonadotropin-releasing hormone; BMD, bone mineral density; IVF, *in vitro* fertilization; MRI, magnetic resonance imaging; NSAIDs, non-steroidal anti-inflammatory drugs; QoL, quality of life; SPRM, selective progesterone receptor modulator; AI, aromatase inhibitor; PDE, phosphodiesterase; RCT; randomized controlled trial; ROS, reactive oxygen species; MMP, matrix metalloproteinase; VEGF, vascular endothelial growth factor; HDAC, histone deacetylase; DNMT, DNA methyltransferase; ICG/NIR, indocyanine green/near-infrared (imaging); HA, hyaluronic acid (adhesion barrier).

**Table II:** Biomarker candidates and maturity.

Biomarker/modality	Description (diagnostic/prognostic potential)	Key limitations	(Refs.)
Imaging (TVUS, MRI)	Standard diagnostic imaging modalities. TVUS is first-line for adnexal/endometrioma detection; MRI is used for detailed mapping of deep and multi-compartment disease.	Operator-dependent; may miss subtle/ superficial lesions; requires standardized protocols (MRI).	(85)
PET-FES (fluoroestradiol PET)	Investigational diagnostic imaging. A pilot study (n=6) found no added lesion detection vs. MRI, indicating limited sensitivity in early data.	Very small sample size; initial results negative; needs larger validation studies.	(86)
MMP-9 (protein)	Candidate diagnostic biomarker: consistently elevated in endometriosis across studies (pooled SMD=1.31). Suggests potential for noninvasive detection of lesions.	High heterogeneity (I²>80%); lack of large validation cohorts; uncertain clinical thresholds.	(88,89)
TIMP-1 (protein)	Candidate biomarker with inconsistent findings: studies report decreased levels in blood/ovary and increased levels in peritoneal fluid in endometriosis.	Menstrual cycle effects; contradictory data across tissues; no agreed direction of change.	(88)
TNF-α and other pro-inflammatory cytokines	Inflammatory markers that are elevated in endometriosis (reflecting local inflammation). Proposed for diagnosis in panels rather than alone.	Low specificity for endometriosis; poor diagnostic accuracy alone; best in combination panels.	(88)
miR-451	Promising diagnostic marker: reproducible elevation across fluids in endometriosis. Performs well when combined with other miRNAs in multi-marker panels.	Variability between studies; requires standardized assays; prognostic value untested.	(88)
cirDNA	Explored as a non-invasive diagnostic test: studies show no significant difference in cirDNA levels between endometriosis patients and controls.	Very limited data (small cohorts); no diagnostic signal in current studies; high variability in controls.	(91)
Metabolomic profiles (blood, urine, etc.)	Exploratory diagnostic biomarkers: Numerous small studies report altered metabolite patterns (e.g. amino acids, lipids) in endometriosis. Indicative of systemic metabolic shifts.	Large number of candidate markers (~1,100 reported); high false-positive risk; requires rigorous validation.	(87,88)

TVUS, transvaginal ultrasound; MRI, magnetic resonance imaging; PET-FES, positron emission tomography, fluoroestradiol mtracer (FES) molecular imaging; MMP-9, matrix metalloproteinase-9; TIMP-1, tissue inhibitor of metalloproteinases-1; TNF-α, tumor necrosis factor-α; miR-451, microRNA-451; cirDNA, circulating cell-free DNA.

**Table III:** Pivotal clinical trials and outcomes.

Therapy/intervention	Mechanism/class	Development stage/trial	Key outcomes/notes	Follow-up	(Refs.)
Elagolix (Orilissa)	Oral GnRH receptor antagonist	FDA-approved (2018); phase 3 RCTs completed (EM-I/EM-II)	Significant reduction in dysmenorrhea and pelvic pain vs. placebo (24-week studies). Add-back reduces hypoestrogenic effects.	24 weeks (primary)	(95,96,109)
Relugolix + E2/NETA (Myfembree)	Oral GnRH antagonist + add-back hormones	FDA-approved (2022); phase 3 (SPIRIT 1/2)	Two 24-week RCTs (SPIRIT 1&2) showed > pain relief vs. placebo; 52-week combined extension (SPIRIT OLE) confirmed sustained efficacy/safety.	24 weeks (primary) 104 weeks (OLE)	(97,104,106)
Linzagolix	Oral GnRH antagonist (partial agonism)	EMA-approved (2022); phase 3 (EDELWEISS)	24-week RCT (with and without add-back) showed significant pain relief. Add-back mitigated bone loss. Longer-term data pending.	24 weeks	(98,109)
Vilaprisan	Oral selective progesterone receptor modulator (SPRM)	Phase 2b (trial terminated)	Phase 2b trial (9 weeks treatment) showed no clinical benefit and development was discontinued due to toxicity concerns.	9 weeks	(57)
Aromatase inhibitors (e.g. letrozole)	Inhibit local estrogen production	Early clinical development (systematic review)	Systematic reviews suggest efficacy in reducing lesions, but limited by menopausal side effects. No large RCTs in endometriosis pain reported.	Variable	(58)
Anti-inflammatory/ antioxidants (metformin, astaxanthin, resveratrol)	Reduce oxidative stress/ inflammation in lesions	Pilot trials/small RCTs in progress	Preliminary RCTs show symptom improvement (e.g. metformin, astaxanthin, resveratrol) but data are limited.	12-24 weeks	(61-64)
Immunotherapy (e.g. immune checkpoint)	Modulate immune response in lesions	Preclinical/conceptual (review)	Emerging concept (no clinical trial data). Preclinical studies suggest immune dysregulation in endometriosis, offering targets.	Not applicable	(111)
AT-04 (Angel Touch device)	Portable alternating magnetic field pain-relief device	Phase 3 RCT initiated (protocol published)	1:1 RCT vs. sham (n≈50): 16 weeks treatment (vs. sham) with 4-week follow-up. Primary endpoint=pain score change at 16 weeks. Secondary: Menstrual and non-menstrual pain, dysmenorrhea, quality of life. No results yet.	16 weeks (treatment)	(6)

N, number of participants; RCT, randomized controlled trial; ABT, add-back therapy (hormonal add-back used with GnRH antagonists); BMD, bone mineral density; FDA, US Food and Drug Administration; EM, Elaris (trial program name: EM-I/EM-II) ASRM, American Society for Reproductive Medicine; AST, astaxanthin.

## Data Availability

Not applicable.
